# Supplementary material for: Comparative genomics provides new insights into the diversity, physiology, and sexuality of the only industrially exploited tremellomycete: Phaffia rhodozyma
Source: BMC Genomics. 2016 Nov 9;17:901. doi: 10.1186/s12864-016-3244-7 (PMC5103461; doi:10.1186/s12864-016-3244-7)
Supplement: Additional file 6: — List of orphan genes with links to PFAM (related to Additional file 1: Table S1). (ZIP 1428 kb) [file 12864_2016_3244_MOESM6_ESM.zip › PFAM_UNIQUE/G00647_P.html]

#                                                                            --- full sequence --- -------------- this domain -------------   hmm coord   ali coord   env coord
  
# target name        accession   tlen query name           accession   qlen   E-value  score  bias   #  of  c-Evalue  i-Evalue  score  bias  from    to  from    to  from    to  acc description of target
  
#------------------- ---------- ----- -------------------- ---------- ----- --------- ------ ----- --- --- --------- --------- ------ ----- ----- ----- ----- ----- ----- ----- ---- ---------------------
  
G00647\_P             -            961 ASD2                 PF08687.6    264       0.1   10.6   3.1   1   3       1.5   9.1e+02   -2.4   0.2    51    94    90   136    53   143 0.63 - Apx/Shroom domain ASD2  
G00647\_P             -            961 ASD2                 PF08687.6    264       0.1   10.6   3.1   2   3   0.00068      0.41    8.6   0.4    17    85   202   275   184   302 0.75 - Apx/Shroom domain ASD2  
G00647\_P             -            961 ASD2                 PF08687.6    264       0.1   10.6   3.1   3   3      0.17        99    0.8   0.0    53   135   549   642   504   653 0.63 - Apx/Shroom domain ASD2
